# The Learning Curve for Pancreaticoduodenectomy: The Experience of a Single Surgeon

**DOI:** 10.3390/life14050549

**Published:** 2024-04-25

**Authors:** Cristian Liviu Cioltean, Adrian Bartoș, Lidia Muntean, Sandu Brânzilă, Ioana Iancu, Cristina Pojoga, Caius Breazu, Iancu Cornel

**Affiliations:** 1 Department of Surgery, “Iuliu Hațieganu” University of Medicine and Pharmacy, 400349 Cluj-Napoca, Romania; cioltean.cristian@umfcluj.ro (C.L.C.); cornel.iancu@umfcluj.ro (I.C.); 2 Department of Surgery, Satu Mare County Emergency Hospital, 440192 Satu Mare, Romania; 3 Department of Surgery, Regional Institute of Gastroenterology and Hepatology, 400162 Cluj-Napoca, Romania; 4 Medicover Hospital, 407062 Cluj-Napoca, Romania; sandubrinzila91@gmail.com (S.B.); iancu.ioana@umfcluj.ro (I.I.); 5 Department of Gastroenterology, “Iuliu Hațieganu” University of Medicine and Pharmacy, 400349 Cluj-Napoca, Romania; lidia.ciobanu@umfcluj.ro; 6 Department of Gastroenterology, Regional Institute of Gastroenterology and Hepatology, 400162 Cluj-Napoca, Romania; cristina.pojoga@ubbcluj.ro; 7 Department of Clinical Psychology and Psychotherapy, Babeș-Bolyai University (UBB Med), 400015 Cluj-Napoca, Romania; 8 Department of ICU, “Iuliu Hațieganu” University of Medicine and Pharmacy, 400349 Cluj-Napoca, Romania; breazu.caius@umfcluj.ro; 9 Department of ICU, Cluj-Napoca County Emergency Hospital, 400006 Cluj-Napoca, Romania

**Keywords:** pancreaticoduodenectomy, learning curve, complications, blood loss, operative time, length of stay

## Abstract

Background and Aims: Pancreaticoduodenectomy (PD) is a complex and high-skill demanding procedure often associated with significant morbidity and mortality. However, the results have improved over the past two decades. However, there is a paucity of research concerning the learning curve for PD. Our aim was to report the outcomes of 100 consecutive PDs representing a single surgeon’s learning curve and to depict the factors that influenced the learning process. Methods: We reviewed the first 121 PDs performed at our academic center (2013–2019) by a single surgeon; 110 were PDs (5 laparoscopic and 105 open) and 11 were total PDs (1 laparoscopic and 10 open). Subsequent statistics was performed on the first 100 PDs, with attention paid to the learning curve and survival rate at 5 years. The data were analyzed comparing the first 50 cases (Group 1) to the last 50 cases (Group 2). Results: The most frequent histopathological tumor type was pancreatic ductal adenocarcinoma (50%). A total of 39% of patients had preoperative biliary drainage and 45% presented with positive biliary cultures. The preferred reconstruction technique included pancreaticogastrostomy (99%), in situ hepaticojejunostomy (70%), and precolic gastro-jejunal anastomosis (88%). Postoperative complications included biliary fistula (1%), pancreatic fistula (8%), pancreatic stump bleeding (4%), and delayed gastric emptying (13%). The mean operative time decreased after the first 50 cases (*p* < 0.001) and blood loss after 60 cases (*p* = 0.046). R1 resections lowered after 25 cases (*p* = 0.025). Vascular resections (17%) did not influence the rate of complications (*p* = 0.8). The survival rate at 5 years for pancreatic adenocarcinoma was 32.93%. Conclusions: Outcomes improve as surgeon experience increases, with proper training being the most important factor for minimizing the impact of the learning curve over the postoperative complications. Analyzing the learning curve from the perspective of a single surgeon is mandatory for accurate statistical results and interpretation.

## 1. Introduction

Pancreaticoduodenectomy (PD) is a very complex surgical intervention which is most often performed for pancreatic cancer, distal common bile duct cancer, or carcinoma of the ampulla of Vater, and it must be carried out in high-volume centers by experienced surgeons. The first PD procedure was described in 1930 by Whipple even if it was performed in 1909 in Germany by Kausch [1], while the first learning curve was described by TP Wright in 1930 and it refers to the characteristics of the surgical act (blood loss, operative time, resection margins, and lymph nodes) and to the postoperative outcome (analgesic necessary, blood transfusion, days of hospital stay in ICU and on the surgical ward, morbidity, mortality, and survival) [2]. It is very hard to define a learning curve in PDs due to the fact that while the surgeons gain more experience, they will accept more difficult cases. It is also described in the literature that PDs are usually surgical interventions which are performed on elderly patients which usually have comorbidities which can influence the postoperative outcome. Birkmeyer et al. showed in a study that patients who underwent PD in a high-volume center had better outcomes and also had a superior 3-year survival than patients who had been operated in low-volume centers [3,4]. Lieberman et al. showed in a study that the mortality rate is about 19% in hospitals where fewer than 10 pancreatic resections are performed every year compared to 6% in hospitals where more than 81 pancreaticoduodenectomies are performed every year [5]. Furthermore, the individual experience of every surgeon plays an important role in better postoperative outcomes for the patients. The presence of a learning curve is suggested by the finding that surgeons with higher surgical experience have superior outcomes compared with surgeons with a lower volume of operations performed. Tseng et al. found that after 60 cases, the surgeon gained experience and showed improvement regarding blood loss, operative time, the length of stay, and the achievement of negative margin resection [2]. There are several publications which showed that there is an improvement over time for every surgeon who has a high volume of PDs performed.

However, over the past 20 years, PD has become a common procedure with low mortality and reasonable morbidity because of the multidisciplinary team involved and due to the optimization of the surgical time. Searching in the literature, we found that while all surgeons improve their skills over their career, most of them obtain proficiency at around 60 PDs [6,7]. We present the experience and learning curve of a oncologic surgeon specialized in Whipple surgery in a tertiary care hospital (Adrian Bartos, “Prof. Dr. Octavian Fodor Regional”, Institute of Gastroenterology and Hepatology, Cluj-Napoca, Romania).

## 2. Methods

In this high-volume PD center, between 2013 and 2019, 121 PD procedures were performed by the same surgeon (A.B.); 110 were PDs (5 laparoscopic and 105 open) and 11 were total PDs (1 laparoscopic and 10 open). The medical records of a single surgeon’s first 100 PDs were reviewed.

Patients who had other pancreatic operations such as distal pancreatectomy, central pancreatectomy, enucleation, palliative procedures, or a laparoscopic approach were excluded to allow for a homogenous study.

Medical information was retrospectively collected from the patients undergoing PD from January 2013 to December 2018. Preoperative medical data included age, gender, comorbidities, body mass index, American Society of Anesthesiologists score (obtained from the anesthesia record), use of neoadjuvant treatment or the need of preoperative biliary decompression (endoscopic, surgical-biliary bypasses, or laparoscopic cholecystostomy), routine laboratory testing, chest radiography, contrast-enhanced computed tomography (CT), or magnetic resonance imaging (MRI) to determine the patient’s eligibility for pancreaticoduodenectomy. EUS was performed only on the patients who had a description of vascular invasion on computed tomography. None of the patients had neoadjuvant chemotherapy, and all patients with a malignant result received chemotherapy.

Operative variables included data such as operative time, estimated blood loss, blood transfusion (obtained from the anesthesia record), pancreatic texture (soft/normal/hard), the type of pancreaticoduodenectomy, vascular resection (wedge resection or segmentary resection) if needed, the type of pancreatic reconstruction (pancreato-jejunostomy or pancreaticogastrostomy), the type of gastro-jejunal reconstruction (transmesocolic or precolic route), the type of pancreatic transection (the use of the ultrasound dissector or cold scalpel), concomitant resections, the diameter of the pancreatic duct and common bile duct measured with an ultrasound probe, and the administration of Sandostatin for the prophylaxis of pancreatic fistula. Postoperative data included the length of stay (the number of days on ICU and the number of days on the surgical ward), the day of the removal of the nasogastric tube, the appearance of any complications, the final pathologic exam recorded as well as margin status and lymph node harvest, and the presence of lymphovascular or perineural invasion. Survival data were collected from the county direction of population records.

Complications such as pancreatic fistula, delayed gastric emptying (DGE), and upper digestive hemorrhage were scored using international classification systems according to Clavien Dindo [8].

Gastroparesis or delayed gastric emptying was defined as a failure to consistently tolerate solid oral intake or the presence of a nasogastric tube on or after postoperative day 10. Postoperative hemorrhage was defined as the need to return to the operating room or a postoperative endoscopic intervention for hemorrhage. Biliary leak was defined as the drainage of any volume of fluid clinically consistent with bile from operatively placed drains. We defined pancreatic fistula using the International Study Group on Pancreatic Fistula definition, namely, a drain output of any measurable volume of fluid on or after postoperative day 3 with an amylase content greater than 3 times the serum amylase level [9,10]. The length of hospital stay was calculated excluding the day of the surgery and the day of discharge.

The data were analyzed comparing the first 50 cases (Group 1) to the last 50 cases (Group 2).

Postoperative care followed a similar pathway in all cases: first, the patients were in the intensive care unit (ICU) and after a few days, they were transferred to the surgical ward. All patients had postoperative chemotherapy.

Pancreaticoduodenectomy was performed in the same manner for all patients, and the surgeon followed a standard operative protocol. The surgical team was standard and was composed of a specialist doctor and two general surgery residents. In the first 3 cases of vascular resection, the head of the clinic was part of the surgical team. The skin incision was midline or Mercedes, and then the Kocher maneuver was performed to establish the resectability of the tumor. Pancreatic parenchyma was cut with the cold scalpel in the first cases and then the surgeon used the ultrasound dissector. In all cases, PD was performed without preserving the pylorus. Pancreatic reconstruction was performed by means of pancreaticogastrostomy, using an external layer of running 3-0 non-absorbable monofilament sutures or with an inner of interrupted sutures completed by using an external layer of running 3-0 non-absorbable monofilament sutures. Pancreato-jejunostomy reconstruction was used for one patient. Bilio-digestive anastomosis was performed in a single layer using Dacril 3-0. The jejunal loop was accessioned in situ, on the place where the duodenum was or transmesocolic. Digestive reconstruction was performed by a gastro-jejunal anastomosis using an interrupted suture, with an precolic jejunal loop. The transmesocolic jejunal loop was used only if the precolic anastomosis was not possible due to the tension-free principle.

Jejunostomy for enteral nutrition was performed using the Witzel technique for patients with vascular resections or for those with a higher risk of developing complications.

Statistical analysis was reported as frequency and percentage; continuous variables were reported as median with range. For the statistical analysis, we used the Cochran–Armitage trend test (for categorical variables) and the Pearson correlation test (for continuous variables). All tests were two-sided, with alpha level set at 0.05 for statistical significance.

## 3. Results

**Figure 1:**
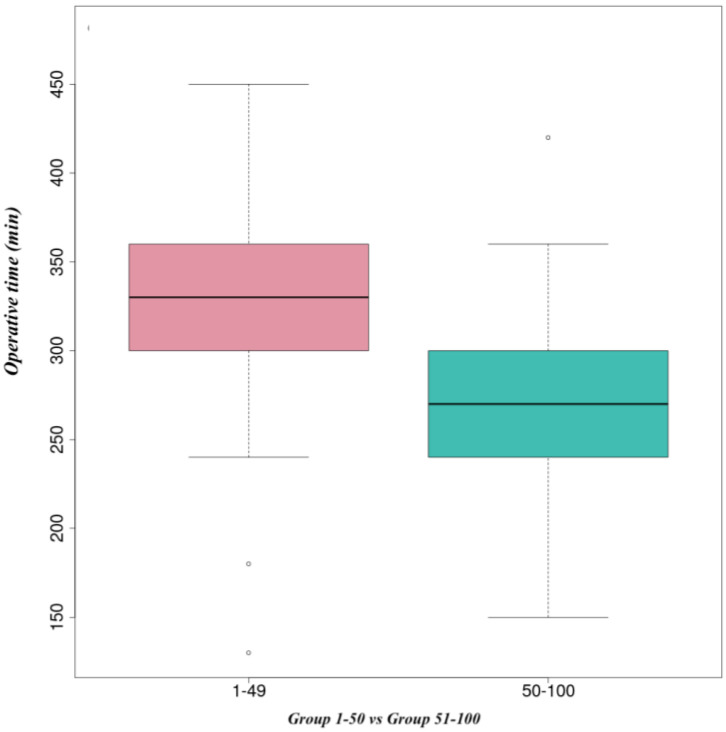
Median time of surgery.

**Figure 2:**
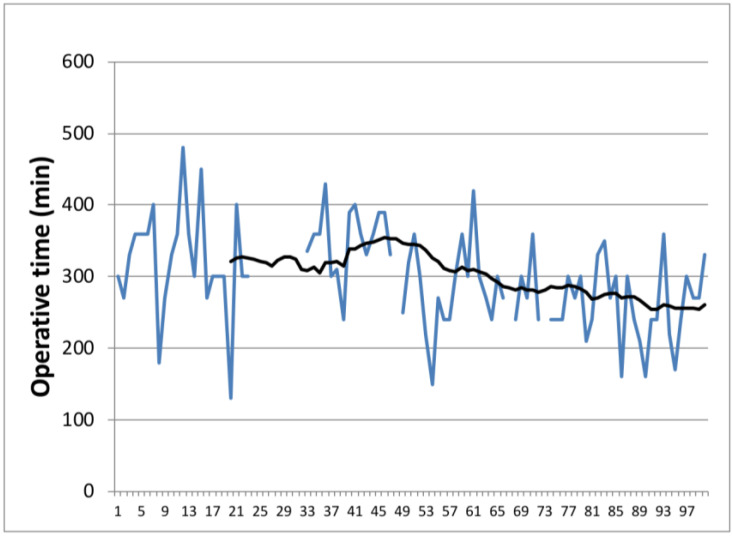
Evolution of duration of the surgery from case 1 to case 100. Color one is the variation of time with each case.

**Figure 3:**
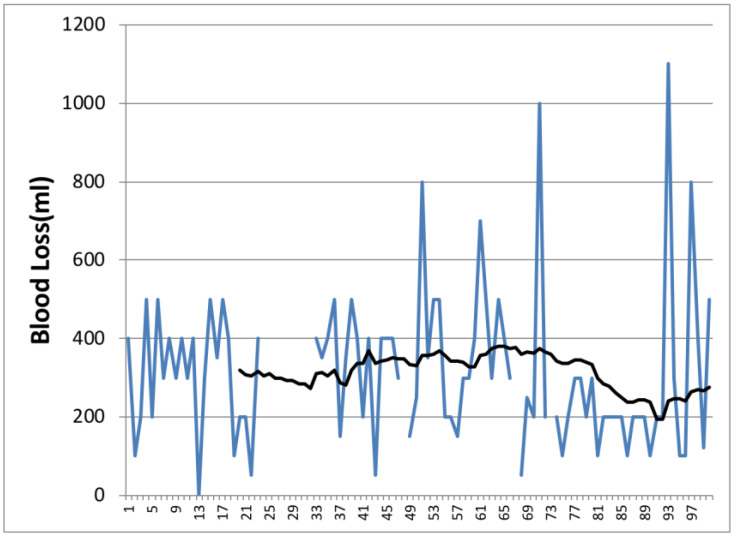
Evolution of the blood loss from the case 1 to case 100. Color one is the variation of time with each case.

**Figure 4:**
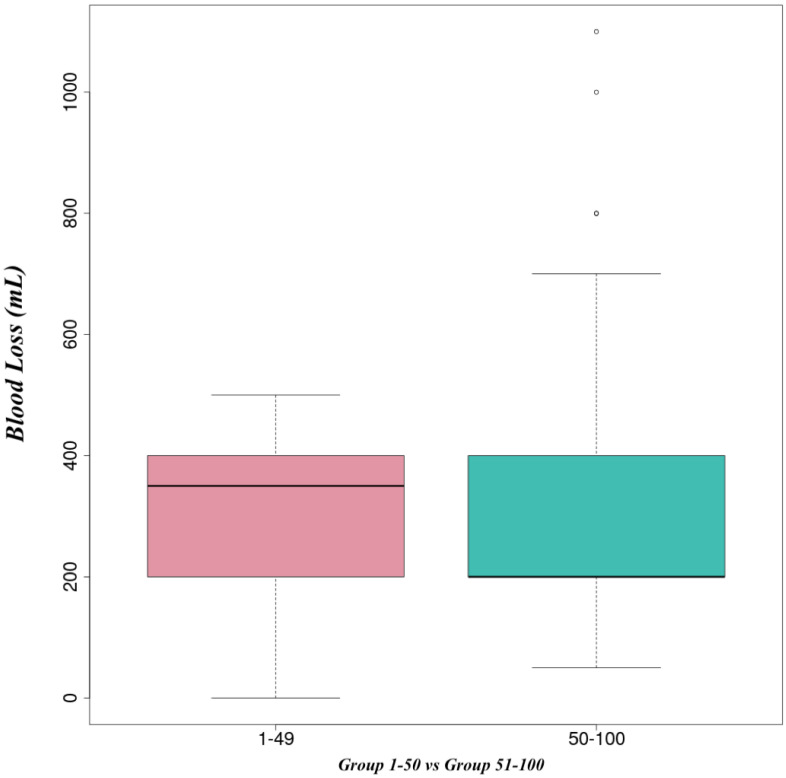
Median of blood loss (comparative between group 1–50 vs. group 51–100).

From 2013 to 2018, we analyzed the first 100 pancreaticoduodenectomies performed by a single surgeon. We found that there were 60 males (25 operated in the first 50 cases) and 40 females (24 operated in the first 50 cases). The median ages of the patients in Groups 1 and 2 were 68 (55–84) and 66 (42–85) years old, respectively. The median operative time decreased from Group 1 where was 333 min to 270 min in the second group, *p* = 0.1 (Figure 1 and Figure 2). The median blood loss was 350 mL in the first group, while in the second group, the median was 200 mL, *p* < 0.001 (Figure 3 and Figure 4). The median length of stay decreased from Group 1 to Group 2, 11 (7–44) versus 10 (7–30) days. A total of 39% of the patients had a preoperative biliary drainage, which was performed endoscopically in 33% of cases and surgically (laparoscopic cholecystostomy) in 6% of cases. A total of 45% of the patients presented with cholangitis (positive biliary cultures—intraoperative bile harvest). We did not find any significant association of complications in patients with positive biliary cultures (*p* = 0.714).

The sectioning of the pancreas was performed with the cold scalpel in the first 32% of cases and then the harmonic scalpel was used (68% of cases).

Vascular resection was performed in 17% of cases; in 9% of cases, a wedge resection was performed while in 8% of cases, a segmentary vascular resection was needed. Vascular resections were not associated with a higher risk in developing postoperative complications (*p* = 0.8).

We found that there is a higher risk for a pancreatic fistula if the pancreatic tissue is soft (*p* = 0.014), but there was no difference if we used a pure string (37%), separate sutures (45%), or both types (18%) in the pancreatic reconstruction (*p* = 0.07). All pancreatic fistulas were Grade B. In our study, we performed 99 pancreaticogastrostomies and 1 pancreato-jejunostomy. Comparing the two groups, the rate of pancreatic fistula was higher in the second group (*p* = 0.017). According to our database, the gastro-jejunal anastomosis was performed precolic in 88% of cases and transmesocolic in 12%, while the biliary reconstruction was performed “in situ” in 70% of patients and transmesocolic in 30% of cases. All surgeries were performed with the assistance of residents or fellows.

Regarding survival rate, we found a survival rate at 3 years of 39.91% for pancreatic adenocarcinoma, while the survival rate at 5 years was 32.93%. When we studied the survival rate at 3 and 5 years between the group of the first 50 patients versus the last 50 patients we found that there is a difference—17.8% vs. 30.7% at 3 years and 10.7% vs. 3.8% at 5 years.

**Figure 5:**
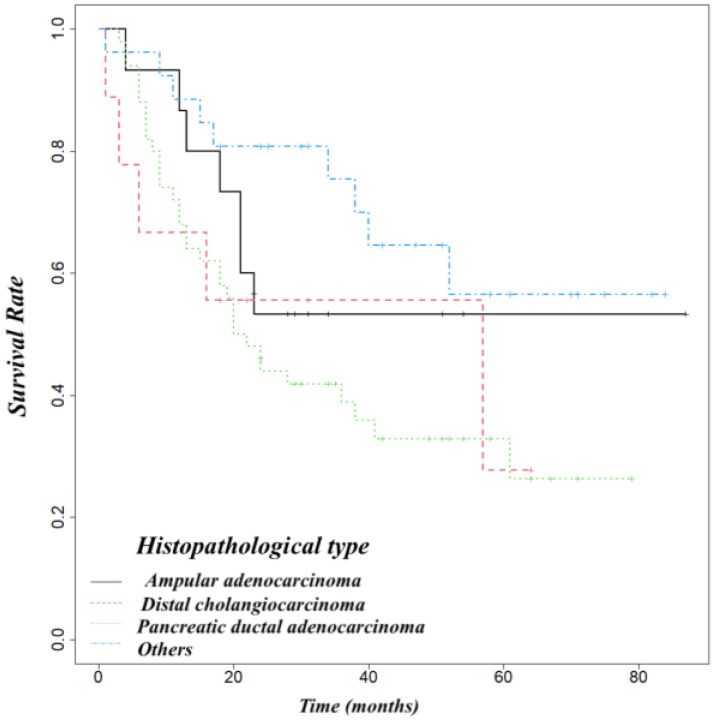
Probability of survival depending on histopathological type.

**Figure 6:**
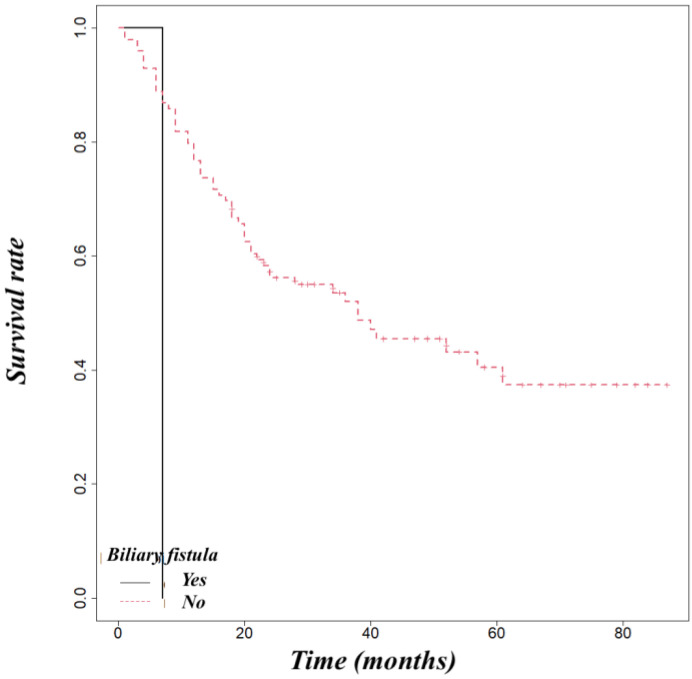
Influence of biliary fistula on survival (*p* = 0.025).

**Figure 7:**
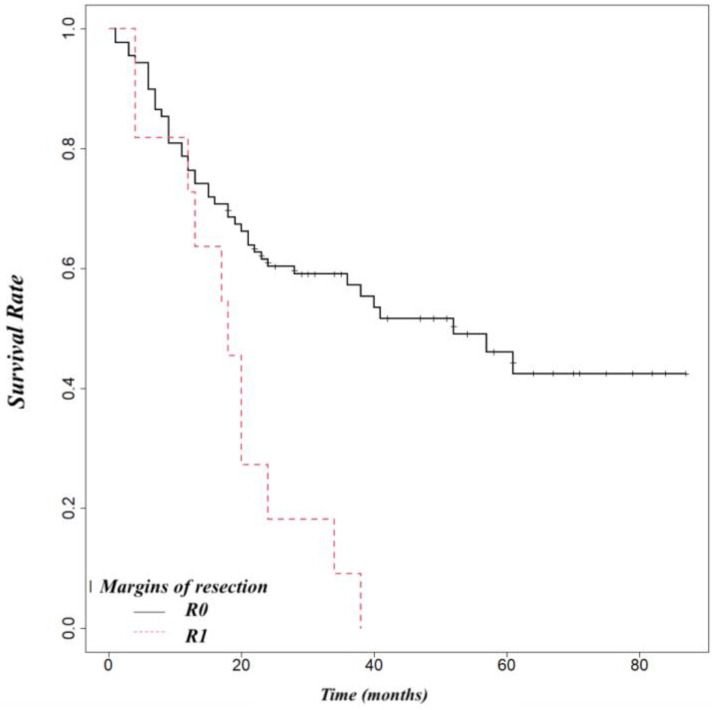
Influence of resection edge-R1 on survival (*p* < 0.001).

**Figure 8:**
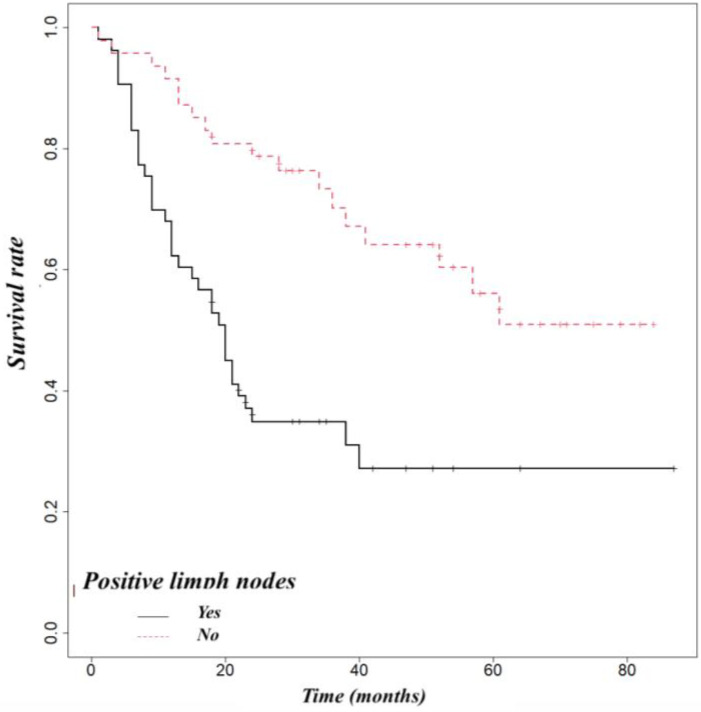
Influence of lymph node metastases on survival (*p* < 0.001).

Pancreatic adenocarcinoma had a worse overall survival than ampullary cancer (OS at 5 year 52%) or distal cholangiocarcinoma (OS at 5 years 54%) (Figure 5). We found that biliary fistula (*p* = 0.025) (Figure 6), resection edge-R1 (*p* < 0.001) (Figure 7), and lymph node metastases (*p* < 0.001) (Figure 8) negatively influenced the survival rate.

Postoperative complications are given in Table 1 and Table 2. There was no significant difference in the appearance of pancreatic fistula or hemorrhage from the pancreatic stump when using the cold scalpel or the harmonic scalpel (*p* = 0.265 and *p* = 1, respectively). We did not find that the administration of Sandostatin has a lower risk of developing pancreatic fistula (*p* = 0.13). Regarding delayed gastric emptying, there was no significant difference in the type of reconstruction used (precolic or transmesocolic) (*p* = 0.653). Vascular resections were not associated with a higher risk of developing postoperative complications (*p* = 0.8) (Table 3 and Table 4).

Most patients underwent pancreatic resection for cancer, and the most frequent type was pancreatic ductal adenocarcinoma (n = 50, 50%) (Tabel 1). For patients with periampullary adenocarcinoma (n = 50, 50%), the size of the primary tumor and number of lymph nodes harvested did not significantly vary over the time period. An R0 resection (negative margins) was obtained in 89% of cases which significantly improved in the last 50 patients (*p* = 0.049).

A separate, similar analysis comparing the first 33 cases to the middle 33 cases and to the last 34 cases revealed similar, significant differences in blood loss (*p* = 0.005), operative time (*p* < 0.001), and the length of stay. Another similar analysis was performed comparing groups of 25 patients. The rate of pancreatic fistula was higher in the last 34 cases (*p* = 0.004). Also, there was a significance rate of complications in the last 25 cases (*p* = 0.042).

## 4. Discussion

Despite the fact that many learning curves have been described for different surgeries, it is very hard to define a learning curve for pancreaticoduodenectomy. The learning curve refers to the characterization of the surgical act (operative time, blood loss, resection margins, and lymph nodes) and the characterization of postoperative outcome (analgesic necessary, transfusion, the length of stay in hospital, morbidity, mortality, and survival). Learning curves may vary and are individual for every surgeon, but may be diminished by appropriate training, high volume practice, and an experienced multidisciplinary team. Also, the outcomes improve as surgeon experience increases, but it must be taken into account that as experience increases, the surgeon will accept more difficult cases.

Pancreatoduodenectomy is the surgical procedure for periampullary tumors and pancreatic head tumors. It is the most complex surgical procedure for surgeons and it is accepted by the oncological hospital that survival could significantly improve when surgical resection is accurate, especially in low stages (I and II) [1,8].

Even though learning curves have been described for many surgical interventions, there are limited data regarding the learning curve for PDs. Even though there are no tips and tricks regarding how to improve the learning curve in a short period of time and how to avoid a learning curve that impacts patient outcomes, surgeons should perform this type of procedure according to oncological requirements [9]. Tseng et al. tried to analyze the learning curve for PD looking at the operative experience of three surgeons. They found that improvements appear after approximately 60 PDs performed [2]. They found an improvement in the blood loss (1100 versus 725 mL), operative time (589 versus 513 min), the length of stay (15 versus 13 days), and the R1 resection rate (30% versus 8%) [2]. They did not study data about morbidity and mortality, but they concluded that after 60 PDs, a surgeon’s surgical technique and postoperative outcomes improve.

Data from the current study confirm the findings of Tseng et al. Similar to Tseng et al., this study showed improvements in operative time (333 min vs. 270 min), blood loss (350 mL vs. 200 mL), and the length of stay with experience (11 vs. 10 days). However, in this study, improvements were seen after 50 cases. This demonstrates that there is a learning curve but it depends on every surgeon due to the individual ability, training volume in preoperative, operative, and postoperative care, the complexity of the cases, and the presence of an experienced multidisciplinary team.

Pancreatic fistula is the most common and serious complication after pancreatic resection and can further cause other complications [10]. Only 8 (8%) clinically relevant pancreatic fistulas (PFs) were noted in the whole cohort, but 7 of them were reported in the group of patients 51–100. This can be explained by the fact that with the acquisition of surgical experience, the surgeon accepted for surgery patients with more advanced disease or with more comorbidities. At the same time, the modification of the surgical technique can contribute to the appearance of this type of complication. We found in the literature that the pancreatic fistula rate was reported by other surgeons in the early stage of their careers. A strategy for performing an excellent pancreaticogastrostomy is also likely important because pancreatic leak is associated with additional complications. At least two modifications in the pancreaticogastrostomy technique were made in this series. Kazanjian et al. showed in a study that the soft pancreas tissue has an increased risk of pancreatic fistula (12.6%) [11]. This is also confirmed by Schimdt et al., who reported an incidence of 9% [12], and by Balcom et al., with an incidence of pancreatic fistula of 11% [13]. Takao et al. conducted a noncomparative clinical study utilizing the harmonic scalpel to transect the pancreas in biliary-pancreatic surgery [14]. They observed that there was no occurrence of pancreatic fistula in 41 patients who received a reconstruction of the remaining pancreas [14]. Suzuki et al. reported in a trial that the pancreatic fistula rate was significantly reduced using ultrasound dissection compared to conventional dissection (3.7% versus 25.8%) [15]. A similar result was also described in the study reported by Sugo et al. [16]. In our study, we found no significant difference in the appearance of pancreatic fistula or hemorrhage from the pancreatic stump when using the cold scalpel or the harmonic scalpel (*p* = 0.265 and *p* = 1, respectively). Even though there are many papers which showed that the administration of somatostatin analogues can lower the risk of developing pancreatic fistula, in our study, we did not find that the administration of Sandostatin is associated with a lower risk of developing pancreatic fistula (*p* = 0.13) [17,18,19,20,21].

Delayed gastric emptying after surgery refers to the condition where the stomach cannot properly take food due to the symptoms of early satiety, nausea, and vomiting following upper gastrointestinal surgery without the mechanical obstruction of anastomosis or distal intestine. Delayed gastric emptying is divided into three grades (A, B, and C) based on nasogastric intubation, the type of diet that the patient is able to intake, the patient’s general health condition, whether a prokinetic drug is used, and the need for further diagnostic tests [8,22,23]. It is one of the most frequent complications after pancreaticoduodenectomy, with an incidence between 7 and 16% [22]. Buchler et al. showed in a study with 331 patients an incidence of delayed gastric emptying of 16.3% [24,25], while Schimdt et al. reported an incidence of delayed gastric emptying of only 7% (n = 516) [26]. For a while, it has been accepted that antecolic reconstruction was associated with a lower incidence of delayed gastric emptying [27,28]. There are some papers which showed that there is no difference regarding delayed gastric emptying between antecolic and retrocolic gastro-jejunal anastomosis [28,29,30,31]. In our study, we performed antecolic gastro-jejunal anastomosis in 88% of patients, and the incidence of delayed gastric emptying was 13%.

Overall, clinically morbidity (Clavien I–V) was noted in 34% (11% vs. 23%) of the entire cohort. Patients from this study spent few days in the ICU and had a short hospital stay. These results are mostly comparable with other papers where outcomes after pancreatoduodenectomy were reported.

In a previous single-surgeon study, 232 PDs were performed with excellent outcomes, and the authors concluded that this provides useful outcome measures from which others can perform a self-assessment to lower complication rates [32,33]. We agree with these authors, who also point out that there are many factors other than surgical experience necessary to obtain optimal outcomes from this complex operation. Surgeon-related factors often focus on technical expertise, but many other factors, such as the decision on whether to operate or not, the judgement regarding the timing, intraoperative decision making regarding the extent of resection, the choice of reconstruction method, and the management of anatomic or disease severity variability, are key components of this expertise. The improvement may be more closely related to a frequency of greater than 11 resections per year. However, no surgeon can start off their practice as an experienced high-volume pancreas surgeon. The learning curve for the Whipple procedure may be so lengthy that it would be extraordinarily difficult to progress completely through it even with an additional year or two of focused fellowship training. Recently, several reports from Europe and the United States demonstrated 5-year survival rates of 25% for pancreatic ductal adenocarcinoma after R0 resection [26,34,35]. Our results are comparable in that we found a 32.93% 5-year survival rate after R0 resection.

One of the most important limitations of this study is represented by the small number of cases (n = 100) and by the examination of a single surgeon’s initial operative outcomes. However, the data from this study present similar outcomes to what Tseng et al. related of a large, single-institution experience with pancreaticoduodenectomies.

## 5. Conclusions

The examination of the learning curve of a single surgeon showed that the blood loss, operative time, and the length of stay are improved after 50 cases, while margin resection (R0 resection) is achieved more often after 25 cases. Vascular resection is not associated with a higher risk of developing complications, while the soft pancreas is associated with a higher risk of developing pancreatic fistula. In our study, we did not find a significant association between delayed gastric emptying and the type of gastro-jejunal anastomosis used.

## Figures and Tables

**Table 1:** Tumor location, complications, and rate of vascular resection.

Tumor Location/Group Patients	Vater’s Ampulla	Distal Common Bile Duct	Pancreatic Head (Adenocarcinoma)	IPMN	Neuroendocrine Tumors	Metastasis from Melanoma	Multicentric Adenocarcinoma	Benign Tumors (Adenoma)/Chronic Pancreatitis
Group 1–100	19 (19%)	10 (10%)	50 (50%)	2 (2%)	8 (8%)	1 (1%)	1 (1%)	9 (9%)
Group 1–50	7 (14%)	4 (8%)	24 (48%)	1 (2%)	1 (2%)	0	0	7 (14%)
Group 51–100	12 (24%)	6 (12%)	26 (52%)	1 (2%)	7 (14%)	1 (2%)	1 (2%)	2 (4%)
Complications/Patient groups	Number % (n = 100)	Group 1–50 (n = 50)	Group 51–100 (n = 50)	*p*-value				
Overall complications (yes vs. no)	34/100 (34)	11 (22.45)	23 (45.1)	0.017				
Pancreatic fistula	8/100 (8)	1 (2.04)	7 (13.73)	0.06				
Biliary leak	1/100 (1)	0 (0)	1 (1.96)	>0.99				
Hemorrhage from pancreatic stump	4/100 (4)	2 (4.08)	2 (3.92)	>0.99				
Delayed gastric emptying	13/100 (13)	5 (10.2)	8 (15.69)	0.415				
Acute pancreatitis	1/100 (1)	0 (0)	1 (1.96)	>0.99				
Wound infection	6/100 (6)	3 (6.12)	3 (5.88)	>0.99				
Pulmonary complications	3/100 (3)	2 (4.08)	1 (1.96)	0.61				
Cardiovascular complications	2/100 (2)	2 (4.08)	0 (0)	0.238				
Other complications	4/100 (4)	1 (2.04)	3 (5.88)	0.618				
Reoperation	1/100 (1)	0 (0)	1 (2)	>0.99				
Vascular resection (total)	16 (16 %)	9 (18%)	7 (14%)					
Wedge resection	8 (8%)	5 (10%	3 (6%)					
Segmentary resection	8 (8%)	4 (8%)	4 (8%)					

IPMN means Intraductal papillary mucinous neoplasms.

**Table 2:** Complications between groups.

Patient Groups	1–25 (n = 25)	25–50 (n = 25)	51–75 (n = 25)	76–100 (n = 25)	*p*-Value
Overall complications (yes vs. no)	8 (33.33)	3 (12)	11 (44)	12 (46.15)	0.042
Pancreatic fistula	1 (4.17)	0 (0)	2 (8)	5 (19.23)	0.71
Biliary leak	0 (0)	0 (0)	1 (4)	0 (0)	0.74
Hemorrhage from pancreatic stump	1 (4.17)	1 (4)	1 (4)	1 (3.85)	1
Delayed gastric emptying	3 (12.5)	2 (8)	4 (16)	4 (15.38)	0.88
Acute pancreatitis	0 (0)	0 (0)	0 (0)	1 (3.85)	1
Wound infection	3 (12.5)	0 (0)	3 (12)	0 (0)	0.06
Pulmonary complications	1 (4.17)	1 (4)	1 (4)	0 (0)	0.707
Cardiovascular complications	1 (4.17)	1 (4)	0 (0)	0 (0)	0.485
Other complications	0 (0)	1 (4)	0 (0)	3 (11.54)	0.189
Reoperation	0 (0)	0 (0)	0 (0)	1 (4)	1

*p* value < 0.05 means that it is statistically relevant.

**Table 3:** Complications associated with vascular segmentary resections.

Vascular Segmentary Resections	Yes (n = 8)	No (n = 92)	*p*-Value
Overall complications (yes vs. no)	1 (12.5)	33 (35.87)	0.259
Pancreatic fistula	0 (0)	8 (8.7)	1
Biliary leak	0 (0)	1 (1.09)	1
Hemorrhage from pancreatic stump	0 (0)	4 (4.35)	1
Delayed gastric emptying	1 (12.5)	12 (13.04)	1
Acute pancreatitis	0 (0)	1 (1.09)	1
Wound infection	0 (0)	6 (6.52)	1
Pulmonary complications	0 (0)	3 (3.26)	1
Cardiovascular complications	0 (0)	2 (2.17)	1
Other complications	0 (0)	4 (4.35)	1

**Table 4:** Complications associated with vascular wedge resections.

Vascular Wedge Resections	Yes (n = 8)	No (n = 92)	*p*-Value
Overall complications (yes vs. no)	4 (50)	30 (32.61)	439
Pancreatic fistula	1 (12.5)	7 (7.61)	0.5
Biliary leak	0 (0)	1 (1.09)	1
Hemorrhage from pancreatic stump	1 (12.5)	3 (3.26)	287
Delayed gastric emptying	1 (12.5)	12 (13.04)	1
Acute pancreatitis	0 (0)	1 (1.09)	1
Wound infection	0 (0)	6 (6.52)	1
Pulmonary complications	1 (12.5)	2 (2.17)	223
Cardiovascular complications	0 (0)	2 (2.17)	1
Other complications	1 (12.5)	3 (3.26)	287

## Data Availability

The data presented in this study are available on request from the corresponding author. The data are not publicly available due to confidentiality reason.
